# Targeting G-quadruplex for rescuing impaired chondrogenesis in WRN-deficient stem cells

**DOI:** 10.1186/s13578-022-00939-8

**Published:** 2022-12-31

**Authors:** Adrian On-Wah Leung, Tsz-Ching Yiu, Lingxiao Liu, Hei-Yin Tam, Shen Gu, Jiajie Tu, Duanqing Pei, Hoi-Hung Cheung

**Affiliations:** 1 Key Laboratory for Regenerative Medicine, Ministry of Education, School of Biomedical Sciences, Faculty of Medicine, The Chinese University of Hong Kong, Hong Kong SAR, China; 2 Centre for Regenerative Medicine and Health, Hong Kong Institute of Science and Innovation, Chinese Academy of Sciences, Hong Kong SAR, China; 3 Institute of Clinical Pharmacology, Key Laboratory of Anti-Inflammatory and Immune Medicine, Ministry of Education, Anhui Collaborative Innovation Center of Anti-Inflammatory and Immune Medicine, Anhui Medical University, Hefei, China; 4 Laboratory of Cell Fate Control, School of Life Sciences, Westlake University, 310024 Hangzhou, China

**Keywords:** WRN, SHOX, G-quadruplex, Chondrogenesis, Short stature, Werner syndrome

## Abstract

**Background:**

Pathogenic mutations in *WRN* are a cause of premature aging disease Werner syndrome (WS). Besides accelerated aging phenotypes and cancer predisposition, patients with WS also display underdevelopment in the skeletal system, characterized by short stature, light body weight and unusually thin extremities. The reasons for these developmental defects are not completely understood and the underlying molecular mechanism remains to be elucidated.

**Results:**

In this study, WRN was found to modulate transcription of *short stature homeobox* gene *SHOX*. Loss of WRN resulted in insufficient expression of SHOX, the gene dose of which is critical for driving chondrocyte differentiation. WRN could bind the G-quadruplex (G4) structures in the *SHOX* promoter and stimulate transcription. Aberrant formation of G4 structures in WRN-deficient cells impeded normal transcription of SHOX, thus resulting in impaired chondrogenesis. Chondrogenesis could be rescued by overexpression of WRN helicase or SHOX, suggesting that SHOX is a downstream target of WRN. Gene editing of the G4 structures in the *SHOX* promoter could increase SHOX expression, therefore rescuing the impaired chondrogenesis in WRN-deficient cells.

**Conclusions:**

Our data suggest that dysgenesis of the developing bone in WS might be caused by SHOX insufficiency. Aberrant formation of G4 structures in *SHOX* promoter suppresses SHOX expression and impairs chondrogenesis. Targeted mutagenesis in the G4 structures enhances SHOX expression and thus providing an opportunity to rescue the chondrogenic defect.

**Supplementary Information:**

The online version contains supplementary material available at 10.1186/s13578-022-00939-8.

## Background

Werner syndrome (WS) is a premature aging disease characterized by early onset of age-related phenotypes, such as greying hair, hair loss, cataracts, premature arteriosclerosis, skin atrophy or ulcer, diabetes, osteoporosis, hypogonadism, and cancer. Most of the phenotypes appear in adults; however, short stature, which is reported in approximately 95% of WS cases, is evident and usually observed before the appearance of other signs and genetic diagnosis [1, 2]. Children with WS have a slow growth rate, light body weight, and unusually thin extremities. Growth spurt is absent during adolescence, and retarded body development is evident before the onset of other premature aging signs [1, 2].

Genetically, WS is caused by recessive mutations in the *WRN* gene, which encodes a DNA exonuclease/helicase essential for DNA synthesis, repair, and recombination [3]. Loss of WRN leads to genomic instability, accelerated telomere attrition, and replicative senescence [4–6]. However, the mechanism through which WRN loss causes short stature and the underlying molecular basis are not clearly elucidated.

We have previously characterized the transcriptomic difference between iPSC-derived, isogenic *WRN*^+/+^ and *WRN*^−/−^ mesenchymal stem cells (MSC). Downregulation of paracrine factors such as HGF accounts for the impaired pro-angiogenic function in *WRN*^−/−^ MSC [7]. Notably, we also found downregulation of *short stature homeobox* (*SHOX*) gene in the WRN-deficient cells. SHOX is implicated in the process of growth plate development and differentiation of chondrocytes [8, 9]. Therefore, we hypothesize that WRN regulates expression of transcription factor(s) critical for bone development. One potential mechanism that WRN regulates transcription is by resolving the G-quadruplex (G4) structures through the DNA helicase activity [10]. G4 is a G-rich DNA sequence that forms non-B-form structures stabilized by G-quartets, a planar array of four Hoogsteen hydrogen-bonded guanines. G4s are commonly found in gene promoters, gene body and transcription termination sites, and their aberrant formation in gene regulatory regions impedes binding of transcription factors and thus suppression of transcription [11, 12]. In this paper, we identified critical G4 structures within the *SHOX* promoter, and demonstrated that WRN modulated transcription of these G4-containing promoter sequences. Consequently, downregulation of *SHOX* in WRN-deficient cells impaired chondrogenesis. Targeting the suppressive G4s by Cas9/CRISPR could stimulate *SHOX* expression and rescued the impaired chondrogenicity. Our results suggested that the short stature phenotype in WS might be caused by SHOX insufficiency as a consequence of the loss of helicase activity which could be rescued by gene editing the suppressive G4 sequences.

## Results

### G4 abundance is associated with loss of WRN helicase

**Fig. 1:**
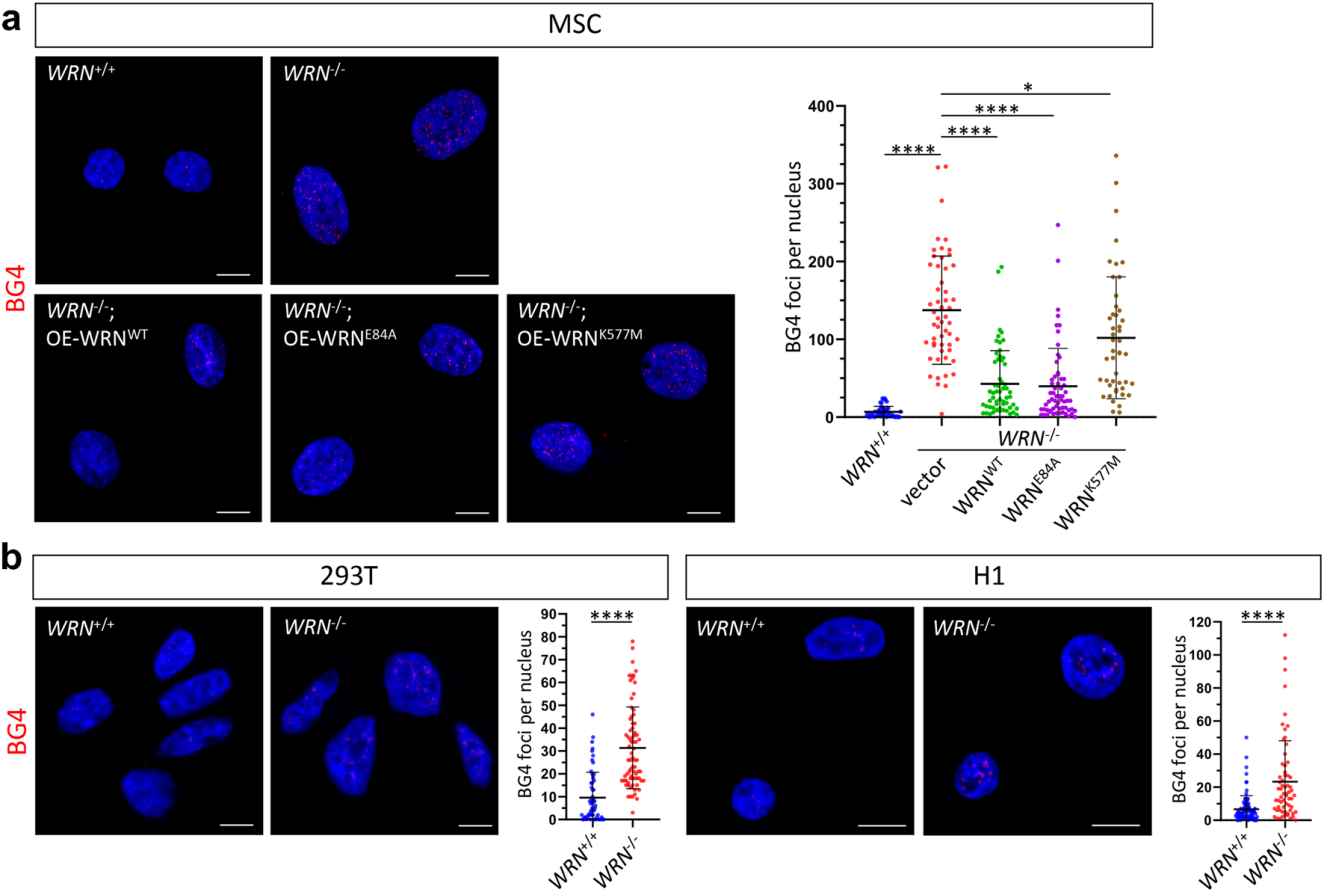
WRN helicase resolves global G4 quadruplexes in multiple cell lines. Representative BG4 staining and quantitative analysis of G4 foci per nucleus in MSC, 293T and H1 cells. N > 47 for each cell type. Scale bar: 10 µm. t-test (2-tailed, unequal variance) compared to *WRN*^−/−^: *p < 0.05, ****p < 0.00005. **a** BG4 staining of *WRN*^+/+^ and *WRN*^−/−^ with OE-WRN in MSC. **b** BG4 staining of *WRN*^+/+^ and *WRN*^−/−^ 293T and H1 cells

WRN protein possesses G4 resolvase activity, thus WRN can bind G4 DNA structures and modulate the transcriptional activity for genes containing G4 structures [10, 13, 14]. However, WRN only binds a distinct subpopulation of G4 motifs in human cells. For instance, the G4 structures of the telomere repeats and *tRNA*, but not *c-MYC* promoter, are the preferred substrates of WRN [15]. To investigate whether loss of WRN results in change of G4 abundance, we stained *WRN*-wildtype (*WRN*^WT^ or *WRN*^+/+^) and *WRN*-knockout (*WRN*^KO^ or *WRN*^−/−^) MSC with BG4, a FLAG-tagged recombinant antibody widely used in detecting a broad spectrum of G4s in vitro and in vivo [16]. We observed a significant increase (*p* < 0.00005) in the number of G4 foci in the nuclei of *WRN*^−/−^ MSC (Fig. 1a). Gene rescue of WRN loss by overexpression (OE) of WT WRN (OE-WRN^WT^), exonuclease-dead (OE-WRN^E84A^) or helicase-dead (OE-WRN^K577M^) mutant proteins revealed the essential role of the helicase activity of WRN in suppressing G4 formation. In *WRN*^−/−^ MSC, the number of G4 foci could be significantly suppressed by OE-WRN^WT^ (*p* < 0.00005) or OE-WRN^E84A^ (*p* < 0.00005), but less significantly by OE-WRN^K577M^ (*p* < 0.05) (Fig. 1a). To determine whether G4 formation is cell-type specific, we also examined G4 abundance in human embryonic kidney cells 293T and human embryonic stem cells (hESC) H1. In line with MSC, WRN loss in both 293T and H1 cells also resulted in global increase of G4 abundance in the nuclei (*p* < 0.00005), suggesting a general role of WRN in resolving nuclear G4 structures (Fig. 1b).

### *SHOX* promoter contains suppressive G4 structures modulated by WRN

**Fig. 2:**
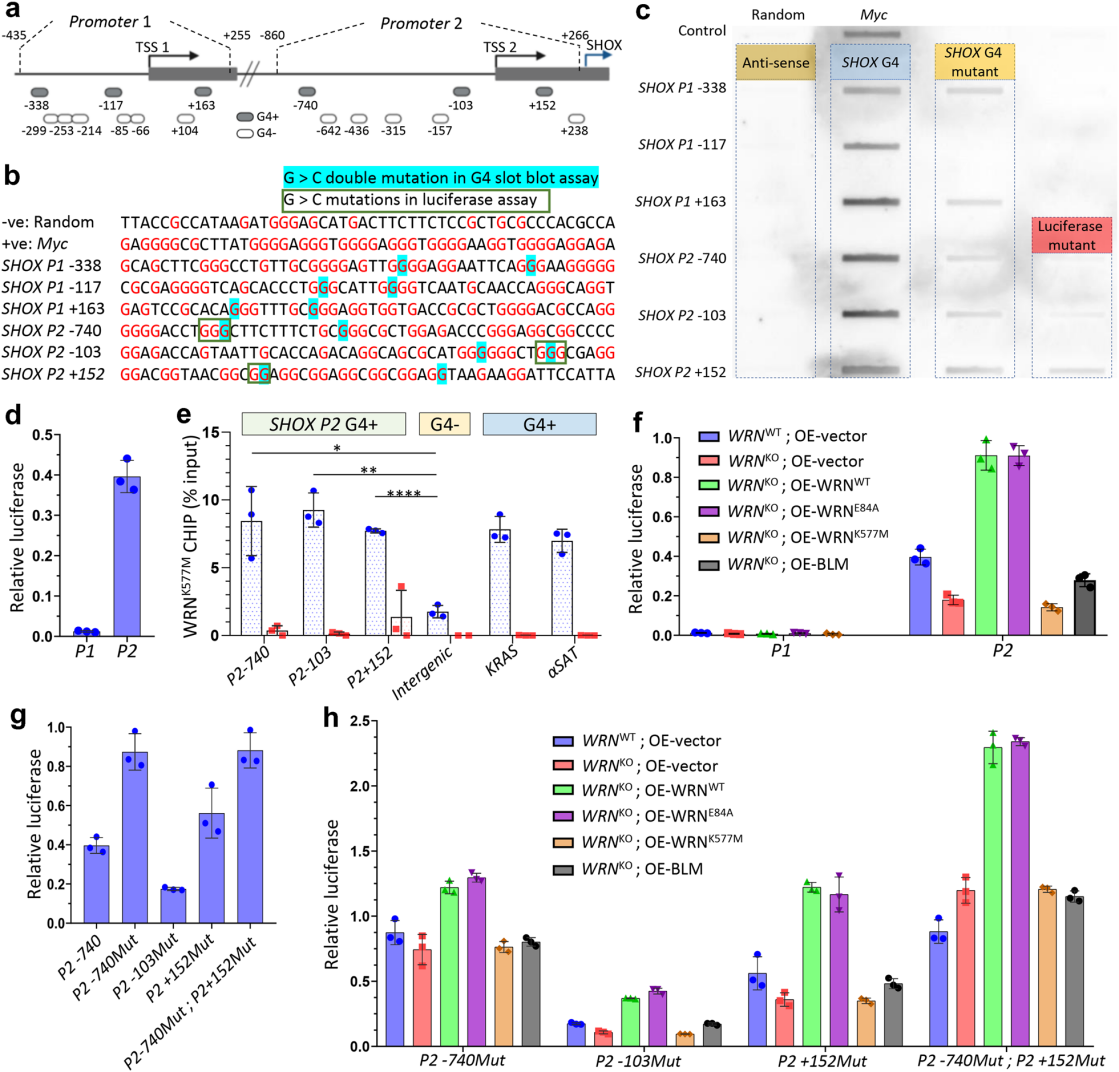
WRN binds to G4 regions of *SHOX* promoter to promote SHOX expression. **a** Illustration of *SHOX* genomic track showing the promoters cloned for luciferase assay in Fig. 1d, g and G4hunter predicted *SHOX* G4 regions based on slot blot assay in Fig. 1c and Additional file 1: Fig. 1. **b** *SHOX* promoter G4s, *MYC* G4 (positive control) and random oligo (negative control) sequences subjected to slot blot assay in Fig. 1c. **c** Slot blot assay of 50 bp ssDNA encompassing G4Hunter predicted G4 regions of *SHOX* promoters. Oligos were heated and gradually cooled to form G4 structures in vitro, followed by nitrocellulose binding and BG4 antibody staining. **d** Luciferase assay of *SHOX* promoters in *WRN*^WT^ 293T. **e** ChIP-qPCR analysis of FLAG-WRN^K577M^ occupancy in *WRN*^KO^ 293 T cells using FLAG antibody for ChIP and PCR primers for amplifying *SHOX P2* promoter G4 regions. Negative control is an intergenic region in Chr6. Positive controls (*KRAS* and *αSAT*) are known binding targets of WRN. N = 3. *t*-test (2-tailed, unequal variance) confidence comparing to G4- intergenic region: *p < 0.05, **p < 0.005, ****p < 0.00005. **f** Luciferase assay of *SHOX* promoters in *WRN*^WT^ and *WRN*^KO^ 293T cells rescued with different WRN mutant proteins. **g** Luciferase assay of *SHOX promoter2* (*P2*) and the different mutated promoters in *WRN*^WT^ 293T. **h** Luciferase assay of mutated *SHOX P2* promoters in *WRN*^WT^ and *WRN*^KO^ 293T cells rescued with different WRN mutant proteins

The human *SHOX* gene contains two alternative promoters (*P1* & *P2*), giving rise to mRNA with different 5′-UTRs but translated to the same polypeptide [17]. In silico analysis by G4Hunter [18] of the *SHOX* promoters identified nine potential/putative G4-forming sequences (PQSs) in *P1* and eight PQSs in *P2*, respectively (Fig. 2a). Slot-blot analysis of each individual PQS confirmed the presence of six G4 structures (three in each promoter) (Fig. 2b, c and Additional file 1: Fig. 1). To further validate the six PQSs as true G4, we synthesized DNA oligos carrying two (G > C) mutations in the G-quartets (G4 mutants), or changed the entire PQSs to antisenses. As expected, the G4 mutants showed reduced G4 formation, whereas G4 formation was abrogated in the antisense oligos (Fig. 2b, c). Next, we examined the promoter activity of *P1* (− 435: + 255) and *P2* (− 860: + 266), respectively. By promoter luciferase activity assay, only *P2* had a strong promoter activity (Fig. 2d). Thus, we focused on the three G4s in *P2*.

Because WRN is a DNA helicase and the binding/dwell time of the enzyme with DNA substrate depends on the enzymatic kinetics, we measured the occupancy of the helicase-dead mutant WRN^K577M^ (which can still bind DNA G4 substrates but is unable to resolve them) at the *P2* G4 sites by chromatin immunoprecipitation followed by quantitative PCR (ChIP-qPCR). The result showed that WRN protein could bind all the three *P2* G4 sites (Fig. 2e and Additional file 1: Fig. 2). To determine whether binding of WRN to *SHOX* promoter promotes transcription, we performed promoter luciferase assay in *WRN*^KO^ cells transfected with rescue plasmids expressing WT or different mutant WRN proteins. As anticipated, both WRN^WT^ and WRN^E84A^ could enhance the luciferase activity, compared with vector control (Fig. 2f). However, WRN^K577M^ failed to stimulate the promoter activity, supporting the notion that the helicase activity is required for resolving the G4 structure in the *SHOX* promoter. We reasoned that if the G4 structures act as transcriptional repressors, partial disruption of G4 structure by (G > C) mutations can eliminate the suppressive effect (Fig. 2b, c). Comparing with WT *P2*, mutations of the − 740 (*P2*-740Mut) and + 52 (*P2* + 152Mut) G4s could increase the promoter activity by 1.5–2.5 folds (Fig. 2g). Promoter that harbored both mutations (*P2*-740Mut;*P2* + 152Mut) could not further increase the promoter activity, which might be due to limited availability of transcriptional factors (Fig. 2g). Similar to WT *P2*, expression of WRN^WT^ or WRN^E84A^ (but not WRN^K577M^) in *WRN*^−/−^ cells could also enhance the mutant *P2* promoter activity (Fig. 2h). Together, these data suggest a role of the WRN helicase in stimulating the *SHOX* expression. Interestingly, expression of BLM, another member of the RecQ helicase family, could not stimulate the *SHOX* promoter activity, nor the G4 mutant promoters (Fig. 2f, h). This additional control suggests that the G4 substrates of WRN and BLM are not completely overlapping.

### WRN loss or SHOX insufficiency impairs chondrogenesis

**Fig. 3:**
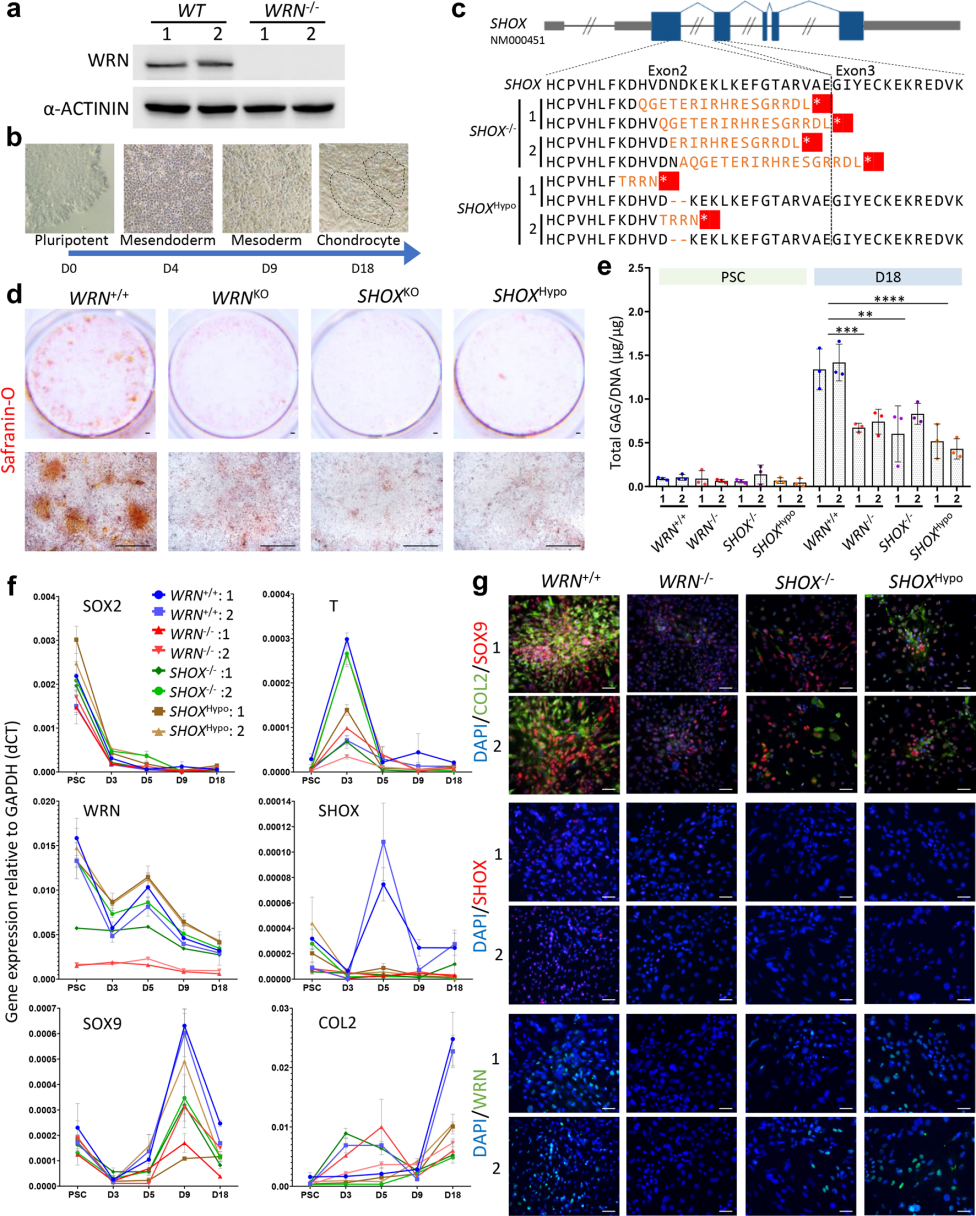
Impaired chondrogenic differentiation in *WRN* KO and *SHOX* KO hESC. **a** Western blot analysis of WRN protein in two *WRN*^−/−^ hESC clones showed null WRN expression. **b** Schematic diagram of directed hESC differentiation to chondrogenic colonies. **c** Multiple alignments for exon 2/3 of WT *SHOX* (NM000451) to *SHOX*^−/−^ and *SHOX*^hypo^ in the gene-edited hESC clones. Variations in protein sequence compared with WT SHOX protein were highlighted in orange. Premature translational terminations were highlighted in red. **d** Safranin-O staining of D18 chondrocytes differentiated from various mutants. N = 3. Scale bar: 500 µm. **e** Quantification of GAG amount in D18 chondrocytes of different mutant cells using DMMB assay. N = 3. *t*-test (2-tailed, unequal variance) on average of both clones compared to WT: **p < 0.005, ***p < 0.0005, ****p < 0.00005. **f** qPCR analysis showing gene expression relative to GAPDH (dCT) of different mutant cells at PSC, D3, D5, D9 and D18 of differentiation. N = 3. **g** Representative images of immunofluorescent staining in D18 chondrocytes. N = 3. Scale bar: 50 µm

Genetic evidences suggest that SHOX plays a critical role in long bone elongation [19]. In human, SHOX is highly expressed in hypertrophic chondrocytes of the growth plate, but not in osteoblasts and osteoclasts [9]. To answer a fundamental question of whether WRN loss causes SHOX insufficiency, we generated two *WRN* KO (*WRN*^−/−^) and two *SHOX* KO (*SHOX*^−/−^) hESC clones by Cas9/CRISPR-mediated gene editing (Fig. 3a, c and Additional file 1: Fig. 2). To model haploinsufficiency of SHOX in causing human short stature, we also generated two hypomorphic *SHOX* (*SHOX*^hypo^) clones (Fig. 3c and Additional file 1: Fig. 3). Directed differentiation of hESC to chondrocytes through mesodermal intermediate was employed [20] (Fig. 3b). Safranin O staining indicated that *WRN*^−/−^ cells were poorly differentiated to chondrocytes. Similar result was observed in *SHOX*^−/−^ cells (Fig. 3d). *SHOX*^hypo^ cells also displayed poor chondrocyte differentiation, indicating that SHOX insufficiency can impair chondrogenesis (Fig. 3d). We also quantitatively measured glycosaminoglycan (GAG) production in chondrocytes, and observed impaired chondrogenesis consistently in all the mutant cells (Fig. 3e). Examination of pluripotency/neuroectoderm marker (SOX2), mesoderm marker (T) and chondrogenesis markers (SOX9, COL2) during chondrocyte differentiation by qPCR revealed a failure to induce SHOX expression at day 5 in both *WRN*^−/−^ and *SHOX*^−/−^ or *SHOX*^hypo^ mutant cells (Fig. 3f). Inductions of SOX9 expression at day 9 and COL2 expression at day 18 were also diminished. Immunofluorescent staining at day 18 also confirmed these differences (Fig. 3g). We also compared osteogenesis in *WRN*^−/−^ hESC, and found impaired osteogenic differentiation as a consequence of WRN loss. Analysis of osteogenic markers revealed downregulation of RUNX2, a transcription factor for osteoblast differentiation (Additional file 1: Fig. 4). As osteogenesis is a subsequent event following chondrogenesis during bone elongation, we focused on chondrogenesis.

**Fig. 4:**
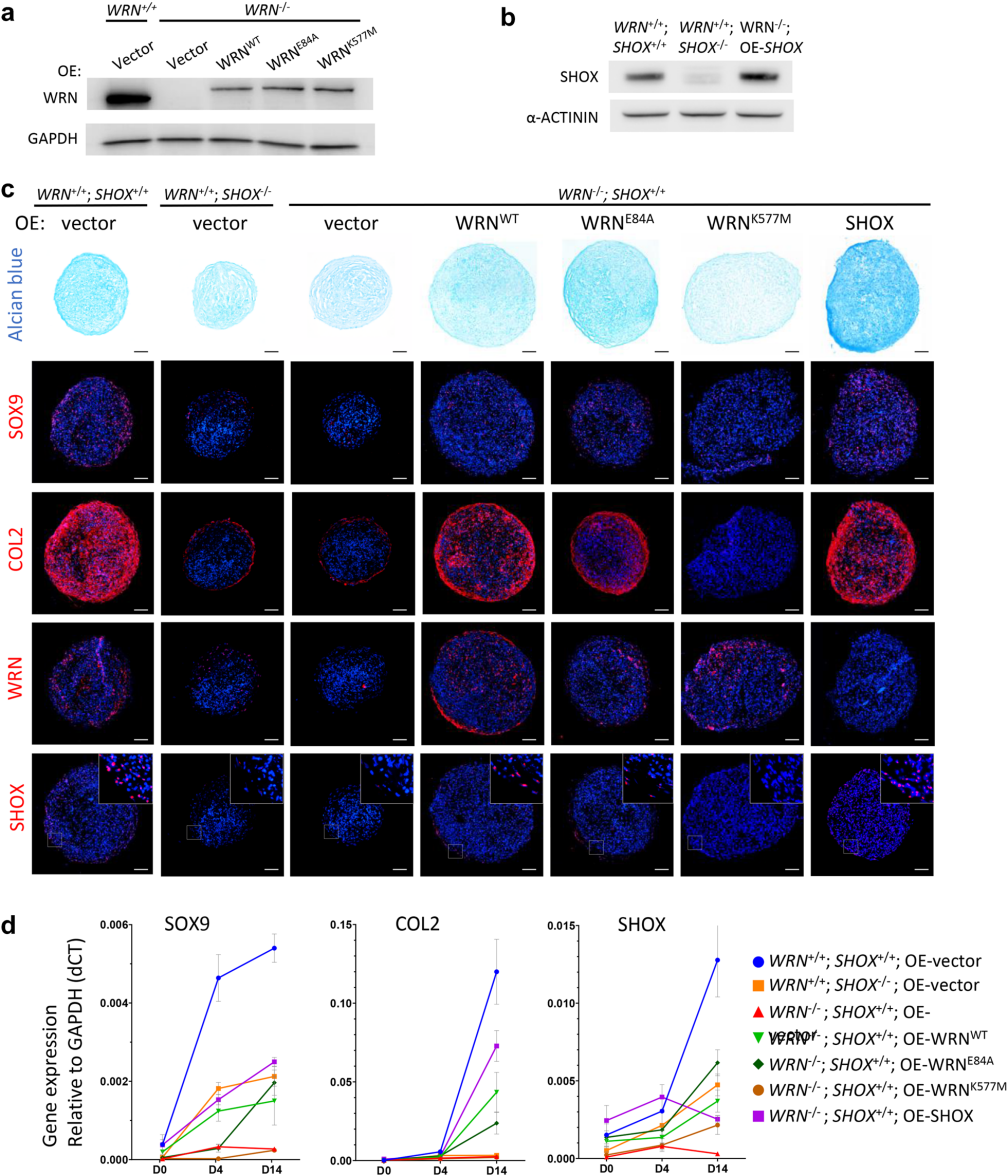
Helicase activity of WRN is required for the rescue of chondrogenic defect in *WRN*^−/−^ MSC by restoring SHOX expression. **a** Western blot analysis of *WRN*^+/+^ and *WRN*^−/−^ MSC rescued by expressions of WT or different mutant forms of WRN proteins. **b** Western blot analysis of MSC as additional controls: *SHOX* KO in *WRN*^+/+^ MSC and *SHOX* OE in *WRN*^−/−^ MSC. **c** Alcian blue staining and immunofluorescent staining for chondrogenic markers (SOX9, COL2) in D14 chondrogenic pellets. Expression of WRN and SHOX proteins were also shown. N = 3. Scale bar: 100 µm. **d** RT-qPCR analysis of SOX9, COL2 and SHOX transcripts at D0, D4 and D14 during chondrogenesis. N = 3

### Chondrogenesis is attenuated in *WRN* or *SHOX* KO MSCs and can be rescued by WRN helicase

WRN is a multifunctional protein with two different enzymatic activities: exonuclease and helicase. As only the helicase is required for stimulating *SHOX* expression, we asked whether the impaired chondrogenesis in *WRN*^−/−^ cells is exonuclease or helicase dependent. To address this question, we performed gene rescue experiments in *WRN*^−/−^ MSC, which was transduced with lentiviruses expressing either WRN^WT^, WRN^E84A^, WRN^K577M^ proteins or vector control (Fig. 4a). As additional controls, we also generated *SHOX* KO in *WRN*^+/+^ MSC (*WRN*^+/+^; *SHOX*^−/−^), or rescued *WRN*^−/−^ cells with SHOX overexpression (*WRN*^−/−^; OE-*SHOX*) (Fig. 4b). Following chondrogenic induction, *SHOX*^−/−^ MSC differentiated poorly, as revealed by weaker Alcian blue staining and smaller chondrocyte cell mass (Fig. 4c). Immunofluorescent staining and RT-qPCR analysis of SOX9 and COL2 mRNAs showed attenuated expression of these chondrogenic markers (Fig. 4c, d). Similar results were observed in *WRN*^−/−^ cells (Fig. 4c, d). Gene rescue of *WRN*^−/−^ by WRN^WT^ or WRN^E84A^ could successfully enhance chondrogenesis as revealed by increased Alcian blue stain and COL2 expression. However, WRN^K577M^ failed to rescue the impaired chondrogenesis (Fig. 4c, d). Gene rescue of *WRN*^−/−^ by SHOX overexpression could also enhance chondrogensis despite the lack of WRN. Together, these results indicate that SHOX is a downstream target of WRN, and the helicase activity of WRN is responsible for modulating SHOX expression.

### Mutagenesis of G4 structures in *SHOX* promoter rescues chondrogenesis

**Fig. 5:**
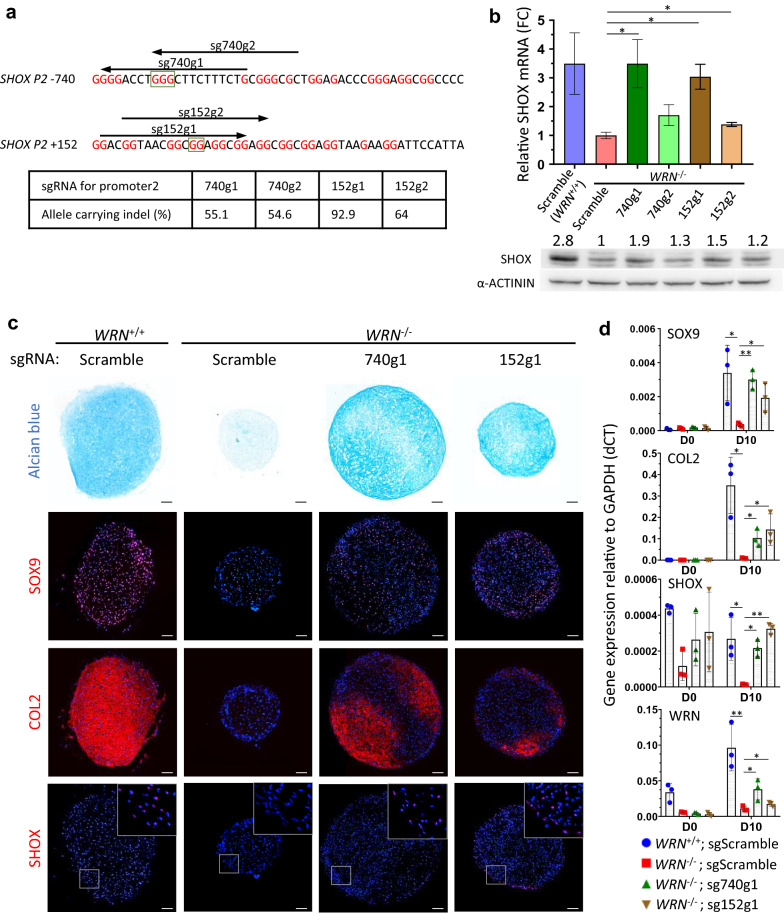
Gene editing of *SHOX* promoter G4s restores SHOX expression and chondrogenicity in *WRN*^−/−^ MSC. **a** Schematic illustration of sgRNAs targeting two G4 sites in *SHOX* promoter. Red: G nucleotides. Box: G > C mutants tested in luciferase assay in Fig. 2c. Indel percentage of sgRNA targeted MSC is calculated by (1- non-indel%) predicted by TIDER. **b** Averaged fold change of SHOX mRNA in G4 gene-edited *WRN*^−/−^ MSC as compared to scramble sgRNA. Error bar: SEM. N = 3. *t*-test confidence (1-tailed, unequal variance) against *WRN*^−/−^: *p < 0.05, **p < 0.005. Western blot confirms elevated SHOX protein level in G4 gene-edited MSC. Number represents relative SHOX protein against *WRN*^−/−^ MSC calculated by intensity of SHOX/ACTININ. **c** Alcian blue and immunofluorescent staining of SOX9, COL2 and SHOX in D10 MSC-differentiated chondrogenic pellets. N = 3. Scale bar: 100 µm. **d** qPCR analysis showing gene expressions of SOX9, COL2, SHOX and WRN relative to GAPDH (dCT) in G4 gene-edited MSC-differentiated chondrocytes at D0 and D10. N = 3. *t*-test confidence (1-tailed, unequal variance) of D10 chondrocytes against *WRN*^−/−^: *p < 0.05, **p < 0.005

Genetic rescue approach by ectopic expression of WRN or SHOX proteins can enhance chondrogenicity attenuated in WRN-deficient cells. However, temporal control of gene dose and ectopic expression in non-cartilage tissues are the major concerns for gene therapy. To substantiate our findings on the determinant G4s in the control of SHOX expression, we explored the possibility of mutating the suppressive G4 structures by Cas9/CRISPR. We designed single guide RNAs (sgRNAs) to target the G4s in *P2*-740 (sg740g1 and sg740g2) and *P2* + 152 (sg152g1 and sg152g2) by Cas9/CRISPR-mediated gene editing. The efficiency of indel formation by these sgRNAs ranged from 55.1–92.9% (Fig. 5a). qPCR analysis of SHOX mRNA showed significant upregulation of SHOX expression. Notably, sg740g1 could enhance SHOX expression in *WRN*^−/−^ MSC to a level comparable to *WRN*^+/+^ MSC (Fig. 5b). Western blot demonstrated an increase of SHOX protein by 1.2–1.9 folds (Fig. 5b). Next, we tested whether G4 gene editing in *WRN*^−/−^ cells can enhance chondrogenesis. Alcian blue staining and SOX9 and COL2 immunofluorescence all indicated an improved chondrogenesis following gene editing (Fig. 5c). qPCR analysis of SOX9 and COL2 mRNAs confirmed the increased expressions of these chondrogenesis markers during differentiation (Fig. 5d). Examination of WRN and SHOX mRNA indicated that mutagenesis of the G4 structures only affected the transcription of SHOX, but not WRN (Fig. 5d). Overall, these results suggest that downregulation of SHOX in WRN-deficient cells was due to the aberrant formation of suppressive G4 structures in the promoter regions. Mutagenesis of the suppressive G4 structures could alleviate the impaired chondrogenesis and thus might be harnessed for gene editing therapy in WS patients for treating short stature dysgenesis.

### Knockdown of *wrn* or *shox* in zebrafish results in skeletal development defect

**Fig. 6:**
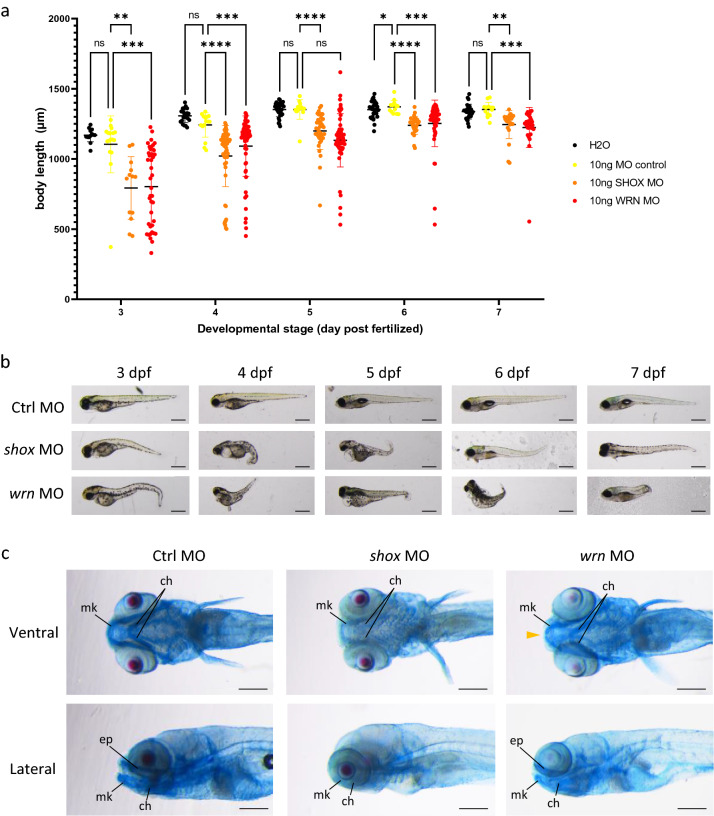
Shortened body length and abnormal cartilage development in *shox* and *wrn* knockdown zebrafish morphants. **a** Quantification of the body length of zebrafish larvae in injection control and morphant groups from 3 to 7 dpf. For each group, n ≥ 13. **b** Representative images of the standard control, *shox* and *wrn* knockdown morphants from 3 to 7 dpf. **c** Ventral and lateral views of the 7 dpf control, *shox* and *wrn* knockdown morphants stained with Alcian blue solution. Some larvae showed defect of skeletal structure including abnormal development of ceratohyal (ch) in *shox* MO larvae and breakage in Meckel’s cartilage (mk) in *wrn* MO larvae (filled arrow). ch, ceratohyal; mk, Meckel’s cartilage; ep, ethomoid plate; MO, Morpholino. Scale bar: 200 µm. *p < 0.05, **p < 0.005, ***p < 0.0005, ****p < 0.00005. *ns* not significant

As both *WRN* and *SHOX* are conserved in zebrafish, we asked whether loss of *wrn* or *shox* in zebrafish causes impaired cartilage development. We examined the phenotypes following knockdowns of these two genes. We injected antisense Morpholinos to the embryos of zebrafish to inhibit *wrn* and *shox* transcripts. Three days post fertilization (dpf), the embryos developed abnormally. Embryos with *wrn* or *shox* knockdown showed shortened body length, compared with wild-type or Morpholino control (Fig. 6a). The knockdown morphants also displayed severe body curvature (Fig. 6b). Whole-mount staining with Alcian blue revealed abnormal cartilage development in these embryos, such as the insufficient formation of ceratohyal and Meckel cartilages (Fig. 6c–h). Together with the human stem cell results, our data suggest that WRN and SHOX play a significant role in skeletal development.

## Discussion

We and others have previously shown that WRN-deficient mesenchymal cells have poor chondrogenic differentiation, which cannot be barely accounted by the increased senescence or reduced proliferation capacity [7, 21]. Notably, downregulation of short stature gene *SHOX* was consistently found in *WRN*^−/−^ MSC. SHOX is a critical transcription factor that regulates chondrocyte differentiation in growth plate and thus controlling long bone development [8, 9]. Mutations of *SHOX* are found in patients with Léri-Weill dyschondrosteosis and Langer dysplasia [22, 23]. Interestingly, mouse *Shox* is absent, and the primary function of *Shox* might be replaced by the paralog *Shox2* [24]. It is intriguing to note that *wrn* mutation or deletion in mouse does not show dwarf or bone degeneration phenotype [25, 26]. However, *wrn* mutation in zebrafish, which retains the *shox* gene orthologue during evolution, does show shorter body length and impaired chondrogenesis [27]. A recent study using zebrafish (*wrn*^−/−^ and *shox*^−/−^) as a model also supports the role of WRN/SHOX axis in bone growth and development [28]. This raises the question of whether WRN/wrn regulates SHOX/shox in a species-dependent manner. In this paper, we demonstrated that the WRN helicase helps to resolve the G4 structures in the promoter of *SHOX*, thus loss of WRN results in insufficient expression of SHOX protein essential for driving chondrogenesis.

G4 structures have been linked to stem cell differentiation and development [29, 30]. The role of G4 in different cellular contexts has been increasingly revealed. For instances, G4 structures at telomeres help to “cap” chromosomal ends, whereas aberrant formation of G4s in the genome hinders DNA replication forks and increases DNA damage leading to genome instability [12]. G4s in gene body are also revealed to change transcription activity, either by inhibiting transcription elongation when G4 structures are present on the template strand, or promoting transcription if G4 structures are formed on the non-template strand. G4s in promoter regions can also change transcription activity, usually by impeding the binding of transcription factors. The three G4 structures identified in *SHOX promoter2* (− 740, − 103, + 152) all showed inhibitory effects on SHOX transcription. Most importantly, expression of wild-type WRN or exonuclease-dead WRN could offset this inhibitory effect. The helicase-dead mutant, however, failed to stimulate G4-containing *SHOX* promoter. These results indicated that the downregulation of SHOX expression in WRN-deficient cells is helicase dependent. Interestingly to note, BLM, another member of the RecQ helicase family implicated in Bloom syndrome, also failed to stimulate *SHOX* promoter activity. In line with the previous analysis on the transcriptional targets by WRN and BLM, many G4-containing genes appear specific to the individual helicases [14]. It might be interesting to delineate the substrate specificity of different G4-resolvases in a genome-wide manner in different cell types.

Lastly, we attempted to rescue the impaired chondrogenesis in WRN-deficient cells by gene editing the G4s without the need of overexpressing WRN or SHOX. This could be achieved by designing sgRNAs that targeted the critical guanine nucleotides in the G4 structures. After gene editing, WRN-deficient MSC could be well differentiated to chondrocytes with improved efficiency, as indicated by enhanced expressions of SHOX, SOX9 and COL2. Clinical trials of gene editing have been successfully conducted and proven effective for treating sickle cell disease and β-thalassemia. Hematopoietic stem and progenitor cells from the patients are gene-edited to reduce BCL11A expression so as to reactivate expression of fetal haemoglobin [31]. Our gene editing result of targeting G4 structures in *SHOX* promoter provides a scientific basis for potentially treating short stature dysgenesis in patients with WS by gene editing approaches.

## Conclusions

**Fig. 7:**
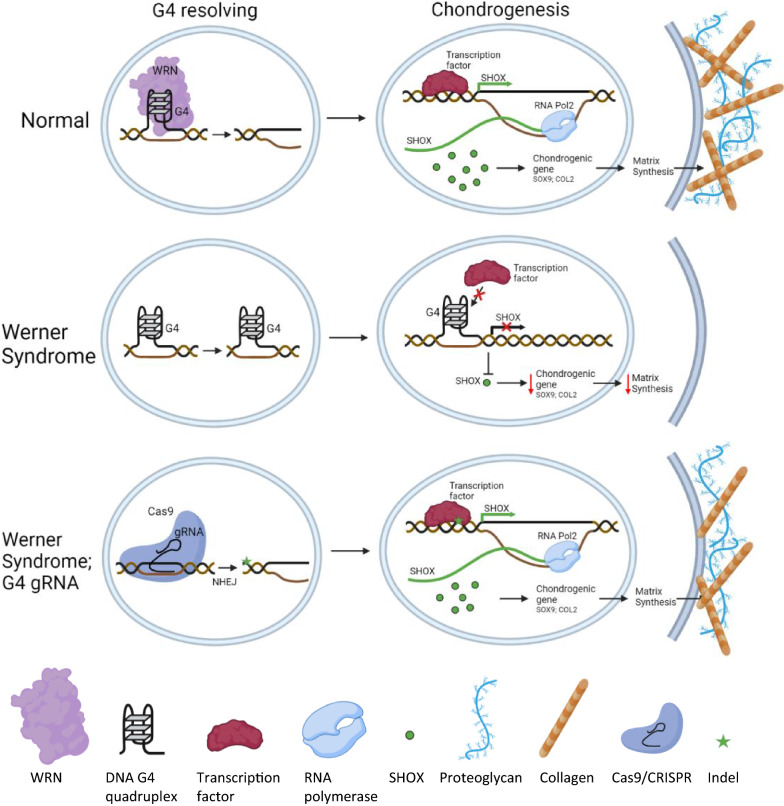
Illustration of G4 retainment in WS causing SHOX insufficiency and chondrogenic defect. Under normal chondrogenesis, WRN resolves *SHOX* promoter G4s to enhance SHOX expression. SHOX then promotes expression of chondrogenic genes to stimulate matrix synthesis. In WS, absence of helicase active WRN leads to aberrant G4s and thus inhibits SHOX expression. Gene rescue by Cas9/CRISPR acting on *SHOX* G4 sequences causes indel and prevents G4 formation. SHOX expression and chondrogenesis are restored in the absence of WRN

To summarize, we showed a molecular mechanism that WRN-deficiency downregulates SHOX expression through modulating the G4 structures in the promoter region. The WRN helicase appears to be responsible for resolving the G4 structures, thus restoration of WRN helicase activity can rescue the impaired chondrogenesis by upregulating SHOX expression. Most significantly, gene editing to the critical G4 sequences can promote chondrogenesis in WRN-deficient cells (Fig. 7).

## Materials and methods

### Plasmid construction

PCR for all cloning was performed with Q5 polymerase (NEB) according to manufacturer’s protocol. Plasmids involving lentiviral components were transformed into Stbl3 *E. coli*, other plasmids into DH5a. All enzymes used were from NEB and all cloning primers and oligos are listed in Additional file 2: Table S1.

Full length *SHOX* promoters were amplified from 293 T genomic DNA using Q5 polymerase and cloned to pGL3-Basic luciferase plasmid (Promega) using SacI;HindIII and MluI;HindIII sites for promoter 1 and 2, respectively. WRN, WRN mutants and BLM expression plasmids (pCMV-WRN/BLM) were obtained as a gift from Dr. Vilhelm Bohr, NIA. Vector control (pCMV-vector) was generated by replacing WRN with linker using NotI;XhoI sites. Mutagenesis on pGL3 containing SHOX P2 promoter was performed using Q5 Site Directed Mutagenesis Kit (NEB) according to manufacturer’s protocol. Full length SHOX was amplified from 293T cDNA and cloned into pHAGE-EF1a-IRES-zsGreen (Addgene #114008) using NotI;BamHI sites, creating pSHOX-GFP.

Mutant MSC and 293T cells were generated using 2-part lentiviral pCas9-Puro and pgRNA-GFP/Cherry system. pCas9-Puro were generated by Gibson assembly using PCR products of primers CP-1 on PX458 (Addgene #48138) and CP-2 on pLenti-TRE3G-BE3RA-PGK-Puro (Addgene #110846) and backbone of ClaI;NotI cut pHAGE-EF1a-IRES-zsGreen. pgRNA-GFP was constructed by inserting a gRNA cassette cloned from pDD-Cas9 (Addgene #90085) into pHAGE-EF1a-IRES-zsGreen with SpeI site. gRNA was inserted by golden gate cloning using BsmBI. All gRNA used in this paper are listed in Additional file 2: Table S2. Lentiviral WRN and WRN mutant overexpression carrying mCherry (pWRN-Cherry) were generated from Gibson assembly of 5 fragments specified in Additional file 2: Table S1 including a backbone fragment cut from NotI;MluI of pLX209-WRN (Addgene #125788). Vector control (pVector-Cherry) was generated by PCR of pWRN-Cherry without WRN sequence and ligating with NdeI site. Cherry version of pgRNA and pSHOX was generated by replacing GFP with mCherry cloned from phSyn-mCherry (Addgene #114472) into NdeI;ClaI cut backbone.

### Culture conditions

Human ESC H1 were maintained in mTESR+ (Stemcell) with normicin on geltrex (Gibco) coating with medium change every 2 days. H1 was passaged by dispase upon 70–80% confluency. MSC were cultured in DMEM (low glucose) medium supplemented with 1 × L-glutamine, 1 × NEAA, 10% MSC-qualified FBS and 1 × PSA (all from Gibco), with medium change every 2 days. MSC was passaged by Tryple Express upon 90% confluent. 293T cells were maintained in DMEM (high glucose) medium supplemented with 1 ×  L-glutamine, 1 ×  NEAA, 1 ×  sodium pyruvate, 10% FBS and 1 ×  PSA (all from Gibco).

### Generation of mutant hESC and chondrogenic differentiation

hESC mutant lines were generated by nucleofection of H1 cells with Cas9/CRISPR PX458 plasmids (Addgene #48138) targeting WRN and SHOX, respectively using Human Stem Cell Nucleofector Kit 1 (Lonza) with A023 program. After nucleofection, GFP-positive cells were FACS sorted and replated in geltrex-coated plates in the presence of ROCK inhibitor Y27632 (Stemcell). Cells were allowed to grow into colonies, expanded and genotyped. The sequences of gRNA used were shown in Additional file 2: Table S2. Chondrogenic differentiation was performed according to published protocol [20]. Since H1 was maintained in feeder-free condition, they were trypsinized to 2.5 × 10^4^/cm^2^ on geltrex coating supplemented with ROCK inhibitor one day prior to differentiation. Other minor modifications included no passage on day12 and extension of differentiation to 18 days to promote chondrogenicity.

### Osteoblast differentiation

Wild-type H1 hESCs and *WRN*^KO^-hESCs were first dissociated by Accutase and seeded at a density of 1 × 10^4^ cells/cm^2^ in osteogenic induction medium for 21 days. Osteogenic induction medium was changed every 2 days and was comprised of 90% knockout DMEM (Gibco), 10% heat-inactivated FBS (Gibco), 1% penicillin-streptomycin (Gibco), 1% GlutaMAX supplement (Gibco), 0.1 μM dexamethasone (Sigma), 100 mM β-glycerophophate (Sigma) and 50 μM ascorbic acid (Sigma). Expression of osteoblast differentiation was determined by alkaline phosphatase, Alizarin Red S staining and qPCR assay on day 7, 14 and 21, respectively.

### Lentivirus production

293T was passaged and seeded in 15 cm dish (14 million cells each). In the next day, second generation lentivirus packaging plasmids were transfected in molar ratio of (Transfer: pMD2G: psPAX2 = 1.5: 1: 1) of 78 µg total DNA with 117 µL of Lipo8000 (Beyotime) in 6.5 ml OPTI-MEM. Lentiviral medium was collected 48 and 72 h post-transfection, filtered with 0.45 µM membrane and addition of PEG8000 (Santa Cruz) to 5% final concentration. After overnight incubation at 4 °C, virus was centrifuged at 1500*g* ×  30 min and the pellet was resuspended in PBS to 50–100X  concentration.

### Gene rescue in MSC by lentiviral transduction

MSC was trypsinized with TrypLE, 0.5 × 10^6^ MSC was incubated with concentrated lentivirus in 8 ug/ml polybrene in 5 ml medium per 10 cm dish overnight. Medium was changed the next day. Control and *SHOX*^−/−^ MSC was generated by first transduced with Vector-Cherry lentivirus. One week later, they were transduced with Cas9-Puro and gRNA-GFP (scramble or SHOX gRNA) lentiviruses. MSC was FACS sorted for gRNA-GFP and puromycin (1 µg /ml) selected for Cas9 for 6 days.

For G4 rescue MSC, they were first transduced with Cas9-Puro lentivirus and selected as above. gRNA-GFP (G4 targeting or scramble gRNA) as well as gRNA-RFP (WRNKO or scramble gRNA) lentiviruses were transduced and FACS sorted for GFP/RFP + cells.

### MSC chondrogenic differentiation

MSC differentiation was performed according to published protocol [32]. Differentiation was initiated in MSC cultured for no more than 17 days post-lentiviral transduction.

### G4 slot blot

Single-stranded DNA (ssDNA) oligos of 50 bp long were purchased commercially. Oligos were diluted to 15 uM in 100 mM KCl to final 200 µL. Diluted oligo of 100 µL per tube was heated at 95 °C for 10 min and gradually cooled to 20 °C at 0.1 °C/sec using PCR machine (C1000 Touch, Bio-Rad). Nitrocellulose membrane (0.45 um, Thermo) and filter papers were pre-wet for 10 min in TBS. After assembly of Slot blot apparatus (1706542, Bio-Rad), 200 µL TBS were applied to each well and vacuumed through. 200 µL of samples or TBS were applied to each well and allowed to slowly pass through membrane on low power vacuum, taking about 10 min to completely drain. Membrane was then briefly dried and baked at 80 °C for 2 h for DNA immobilization. Membrane was then blocked with 10% horse serum, 3% BSA in TBS for 1 h, followed by incubations with BG4 antibody (1:1000) for 1.5 h at RT, anti-FLAG antibody (1:1000) overnight at 4 °C and anti-mouse HRP (1:2500) for 1 h at RT. Membrane was washed 3 times after each antibody incubation with 0.5% Tween/PBS. Antibodies were diluted in 0.5% Tween/PBS. ECL substrate (Bio-Rad) were applied to membrane and exposed with ChemiDoc (Bio-Rad).

### Luciferase assay

293T cells were passaged 1 × 10^5^ per well in 48-well plate. In the following day, medium was changed and 0.6 µg of overexpression plasmids (pCMV-WRN/BLM, a gift from Dr. Vilhelm Bohr, NIA) were transfected using 0.8 µL Lipo8000 (Beyotime) in each well. After overnight incubation, medium was changed and transfected with 0.4 µg pGL3-basic luciferase plasmid (Promega) containing SHOX promoter sequences, 0.04 µg pRL-TK renilla plasmid and 0.6 µL Lipo8000 per well. Next day, cells were digested using Dual-Glo Luciferase Assay System (Promega) according to manufacturer’s protocol. Luciferase and renilla reading were taken by SpectraMax i3x (Molecular Devices) plate reader.

### 293 T mutant generation and ChIP-qPCR

293T cells for ChIP assay was transduced with Cas9-Puro and gRNA-GFP (WRNeij gRNA) lentiviruses then FACS sorted, same method as MSC transduction. These WRN^KO^ 293T were then seeded on 10 cm dish and transfected with pCMV-FLAG-WRN^K577M^ using Lipo8000 (Beyotime). Cells were harvested 2 days post-transfection for ChIP. ChIP protocol was performed according to published protocol [33]. Briefly, 1 × 10^7^ 293T cells were fixed with 1% PFA on dish for 10 min at RT. Then cells were quenched with glycine and chromatin extracted using Chromatrap Sonication Shearing Kit (Chromatrap) according to manufacturer’s protocol. Chromatin was sonicated with SLPe sonicator (Branson) with 2.4 mm tip diameter in 60 V (on:10 s, off:60 s) for 11 min on ice. Sonicated chromatin was diluted tenfold, 30 µL was stored as input while 900 µL were immunoprecipitated with 50 µL FLAG-beads (Sigma) or IGG-beads (CST). After overnight incubation at 4 °C, bound DNA was washed and eluted. Input and precipitated DNA were purified by phenol/chloroform and ethanol precipitation method. qPCR details were shown in Reverse transcription-qPCR section.

### Reverse transcription-qPCR

Cells were flash frozen and later lysed in Trizol (Invitrogen) according to manufacturer’s protocol. MSC-derived chondrogenic pellets were mechanically disrupted with pestles in 1.5 ml tube containing Trizol. RNA was collected by standard isopropanol precipitation. Reverse transcription was performed with Primescript RT master mix (Takara) according to manufacturer’s protocol. Quantitative PCR was performed using Powertrack SYBR MM (Invitrogen) in ABI QuantStudio 7 Real-time PCR System. Fast programme for cDNA samples and standard programme for CHIP DNA samples. Technical triplicate was performed on each sample. All primers for qPCR were listed in Additional file 2: Table S3.

### Western blot

Cells were lysed in RIPA lysis buffer (Beyotime) for 20 min at 4 °C. After centrifuge, supernatant of 20 µg total protein was diluted with 4X Laemmli buffer (Bio-Rad) and denatured at 100 °C for 10 min. Semi-dry transfer was performed with Trans Blot Turbo (Bio-Rad) using standard programme to 0.22 µM membrane. Membrane was blocked with 5% goat serum for 1 h at RT then in respective primary antibody overnight at 4 °C. In the following day, secondary HRP antibody was incubated for 1 h at RT. Washes (3 times) after each antibody incubation step were performed with 0.1% Tween in PBS. All antibodies were diluted in 5% goat serum and concentrations were listed in Additional file 2: Table S4. ECL substrate (Bio-Rad) were applied to membrane and exposed with ChemiDoc (Bio-Rad).

### Tissue processing, immunofluorescence and histological staining

MSC derived chondrogenic pellets and cells were fixed in 4% PFA overnight and 10 min, respectively. Fixed pellets were immersed in 30% sucrose overnight, embedded in OCT and sectioned at 8 µm thickness with cryostat CM3050 (Leica). Pellet sections were pre-treated with 1 mg/ml Pronase E (Sigma) in 37 °C for 5 min prior to staining. Cells and sections were treated with 0.5% Triton ×  for 10 min prior to blocking with 10% goat serum for 1 h. Antibodies were diluted in 5% goat serum with 0.1% Tween-20 according to Additional file 2: Table S4. Primary antibody was incubated O/N at 4 °C and secondary antibody for 1 h at RT. DAPI (1 mg/ml) was incubated for 10 min RT. Prolong glass mountant was used according to manufacturer’s protocol. Fluorescence was imaged by either Ti-2E fluorescence microscope (Nikon) or FV1200 confocal microscope (Olympus). Same staining and imaging criteria were applied between mutants or groups. Images were adjusted by the same LUTs or brightness/contrast among comparing groups.

For histological stain, pellet section or fixed cells were wash with PBS and water, twice each. Cells were then incubated in 0.1% safranin-O solution (Milipore) for 10 min and washed with water for 5 times. Sections were incubated in 1% Alcian blue solution (Milipore) for 20 min RT. Sections were rinsed gently under tap water for 2 min, air-dried and dipped in xylene before mounting in resin mounting medium (Thermo). Histological staining was imaged by IX83 inverted microscope (Olympus).

### DMMB GAG quantitative assay

GAG quantification was performed with DMMB dye according to published protocol [34]. Briefly, samples were digested in 0.15 µg/µL Proteinase K (Thermo) in ammonium acetate buffer (PKAA) at 60 °C for 3 h. Shark chondroitin sulfate (Sigma) (0–1.5 µg) and calf thymus DNA (Sigma) (0–800 ng) were diluted in PKAA as standards. Samples were diluted 5- or 20-fold depending on GAG and DNA content. 200 µL DMMB (sigma) solution (0.016 mg/ml, pH 3) was added to each 20 µL sample, and OD525 measured immediately. 100 µL Hoechst 33258 (Sigma) (0.7 µg/ml) was added to every 100 µL samples and standards. Fluorescence measured with excitation: 340 nm; emission: 465 nm. All readouts were in taken with SpectraMax i3x (Molecular Devices) plate reader and with technical triplicate.

### Statistics and BG4 foci counting

All statistics were calculated with MS Excel using 1 or 2-tailed Student’s t-test assuming unequal variance. Z-stacks of BG4 nuclei staining was maximum projected, brightness adjusted and set an equal threshold to all images. BG4 signals per DAPI were determined by ImageJ “Analyse particle” function with size of “0.01-infinity” with no circularity limitation.

### Injection of Morpholino into zebrafish embryo and whole-mount staining

The use of zebrafish and animal experiments were approved by Animal Experimentation Ethics Committee of CUHK. Wild-type zebrafish (AB line) were maintained at 28.5 °C on a 14 h: 10 h light-dark cycle. Morpholinos (Gene Tools) were injected into the yolks of zebrafish embryos (10 ng/embryo) at one-cell stage using a gas-driven microinjector. The efficiency of Morpholino was visualized by the lissamine conjugated in Morpholino and the expressions of *shox* and *wrn* transcripts were measured by RT-qPCR and phenotypic penetrance. The injected embryos were cultured in E3 embryo media until 7dpf. Body length of each morphant was measured daily from 3 dpf. Morpholinos used in this study are: *shox* MO 5′-AGCGTGCAGAAGAAACTCACCGTCA -3′, *wrn* MO 5′-TTCCTGATGTCTGTGAAAACATATA-3′, and control MO 5′-CCTCTTACCTCAGTTACAATTTTATA-3′. For whole-mount staining, zebrafish embryos at 7dpf were collected and fixed in 10% formalin overnight. Cartilage was stained with Alcian blue solution. The morphants and controls were observed and imaged under Nikon SMZ800 stereo microscope and body length were measured by NIS element.

## Supplementary Information

**Additional file 1: Figure S1.** BG4 slot blot of computational predicted *SHOX* promoter G4 oligos. Slot blot assay of 50bp ssDNA encompossing G4Hunter predicter G4 region of *SHOX* promoters. Oligos were heated and gradually cooled to form G4 structures in vitro, followed by nitrocellulose binding and BG4 antibody staining. **Figure S2.** CHIP-qPCR analysis of WRNWT occupancy in 293T CHIP-qPCR analysis of WRN-/-; CMV-FLAG-WRNWT 293T cells using FLAG antibody targeting SHOX P2 promoter G4 regions. Positive controls are known G4 regions in KRAS and αSAT. n=3. **Figure S3.** Sequencing results of H1 *SHOX* clones. Sequencing chromatogram results of H1 *SHOX* mutant clones and plasmid containing SHOX PCR fragments of the clones showing mutations at gRNA site. **Figure S4.** Osteogenesis of WRN+/+ and WRN-/- cells. WRN+/+ (WT) and WRN-/- (KO) hESCs were induced to differentiation to osteoblasts. Cells were characterized by (a) alkaline phosphatase staining, (b) Alizarin Red S staining at days 7, 14 and 21, respectively. (c) The expressions of osteogenic markers COL1A1, OCN, RUNX2 and OSX by RTqPCR.

**Additional file 2.** Supplementary table containing T1 (primers used in the construction of plasmids), T2 (gRNA sequences), T3 (qPCR primer sequences) and T4 (antibodies used in this study).

## Data Availability

The datasets during and/or analysed during the current study available from the corresponding author on reasonable request.

## Abbreviations

BSA: Bovine serum albumin
ChIP: Chromatin immunoprecipitation
dCT: Delta-CT
DMMB: Dimethylmethylene blue
FBS: Fetal bovine serum
FC: Fold change
G4: G-quadruplex
GAG: Glycosamino glycan
hESC: Human embryonic stem cell
IF: Immunofluorescence
KO: Knockout
mins: Minute
MSC: Mesenchymal stem cell
NEAA: Non-essential amino acid
OE: Overexpression
qPCR: Quantitative PCR
RT: Room temperature
sgRNA: Single guide RNA
WS: Werner syndrome
WT: Wild-type
