# Supplementary material for: Targeting G-quadruplex for rescuing impaired chondrogenesis in WRN-deficient stem cells
Source: Cell Biosci. 2022 Dec 31;12:212. doi: 10.1186/s13578-022-00939-8 (PMC9805690; doi:10.1186/s13578-022-00939-8)
Supplement: Supplementary file 1 — Additional file 1: Figure S1. BG4 slot blot of computational predicted SHOX promoter G4 oligos. Slot blot assay of 50bp ssDNA encompossing G4Hunter predicter G4 region of SHOX promoters. Oligos were heated and gradually cooled to form G4 structures in vitro, followed by nitrocellulose binding and BG4 antibody staining. Figure S2. CHIP-qPCR analysis of WRNWT occupancy in 293T CHIP-qPCR analysis of WRN-/-; CMV-FLAG-WRNWT 293T cells using FLAG antibody targeting SHOX P2 promoter G4 regions. Positive controls are known G4 regions in KRAS and αSAT. n=3. Figure S3. Sequencing results of H1 SHOX clones. Sequencing chromatogram results of H1 SHOX mutant clones and plasmid containing SHOX PCR fragments of the clones showing mutations at gRNA site. Figure S4. Osteogenesis of WRN+/+ and WRN-/- cells. WRN+/+ (WT) and WRN-/- (KO) hESCs were induced to differentiation to osteoblasts. Cells were characterized by (a) alkaline phosphatase staining, (b) Alizarin Red S staining at days 7, 14 and 21, respectively. (c) The expressions of osteogenic markers COL1A1, OCN, RUNX2 and OSX by RTqPCR. [file 13578_2022_939_MOESM1_ESM.pdf]

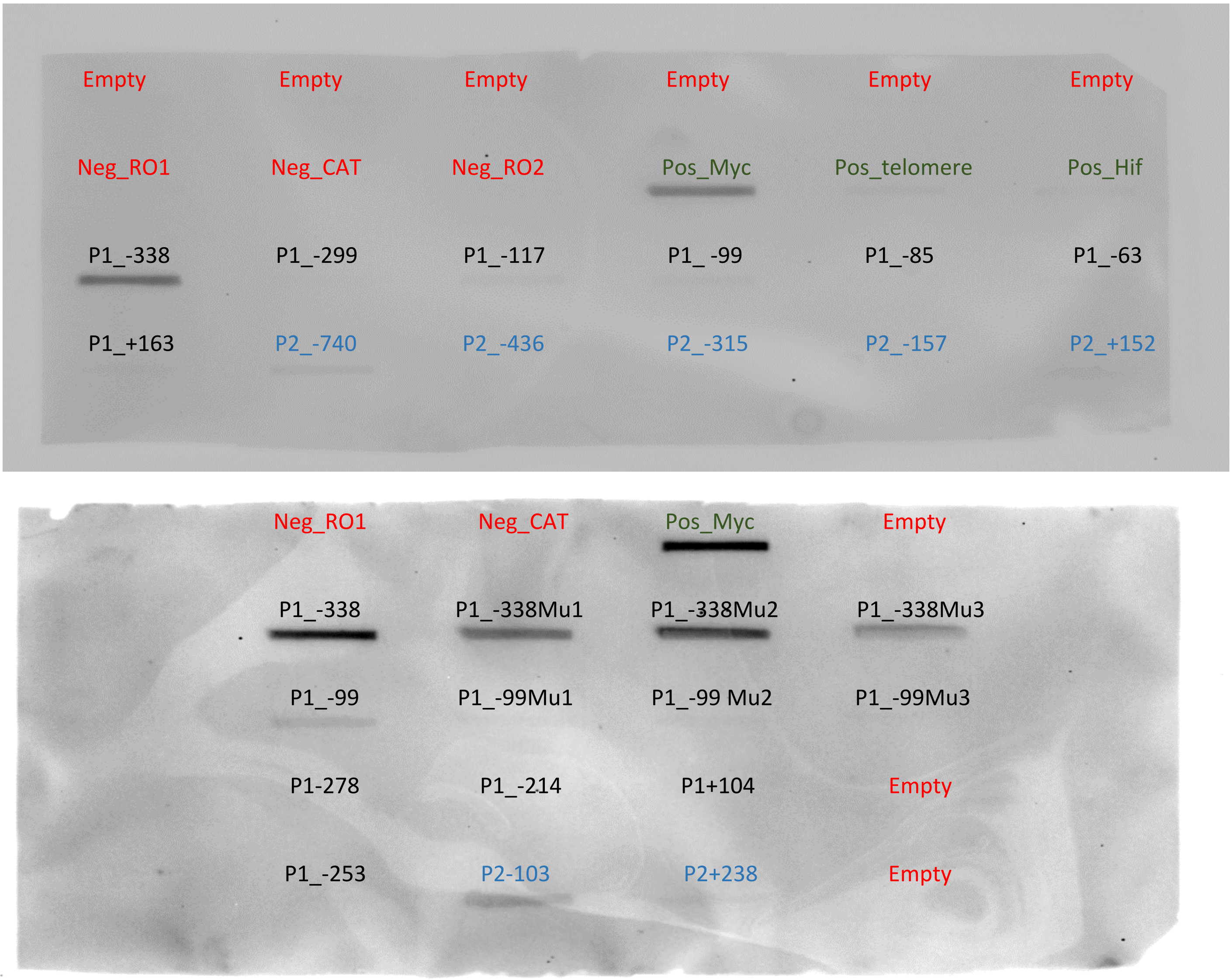

Empty / negative control
Positive control
SHOX Promoter 1 G4 oligo
SHOX Promoter2 G4 oligo

Additional file 1: Figure S1. BG4 slot blot of computational predicted *SHOX* promoter G4 oligos.
Slot blot assay of 50bp ssDNA encompassing G4Hunter predicted G4 region of *SHOX* promoters. Oligos were heated and gradually cooled to form G4 structures *in vitro*, followed by nitrocellulose binding and BG4 antibody staining.

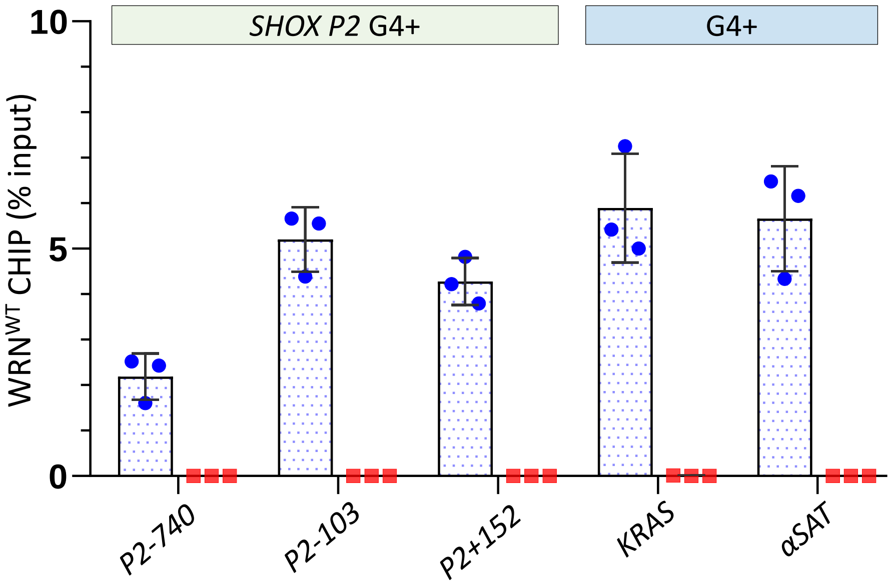

Additional file 1: Figure S2. CHIP-qPCR analysis of WRN^{WT} occupancy in 293T

CHIP-qPCR analysis of *WRN*$^{-/-}$; CMV-FLAG-WRN^{WT} 293T cells using FLAG antibody targeting *SHOX P2* promoter G4 regions. Positive controls are known G4 regions in *KRAS* and *αSAT*. n=3.

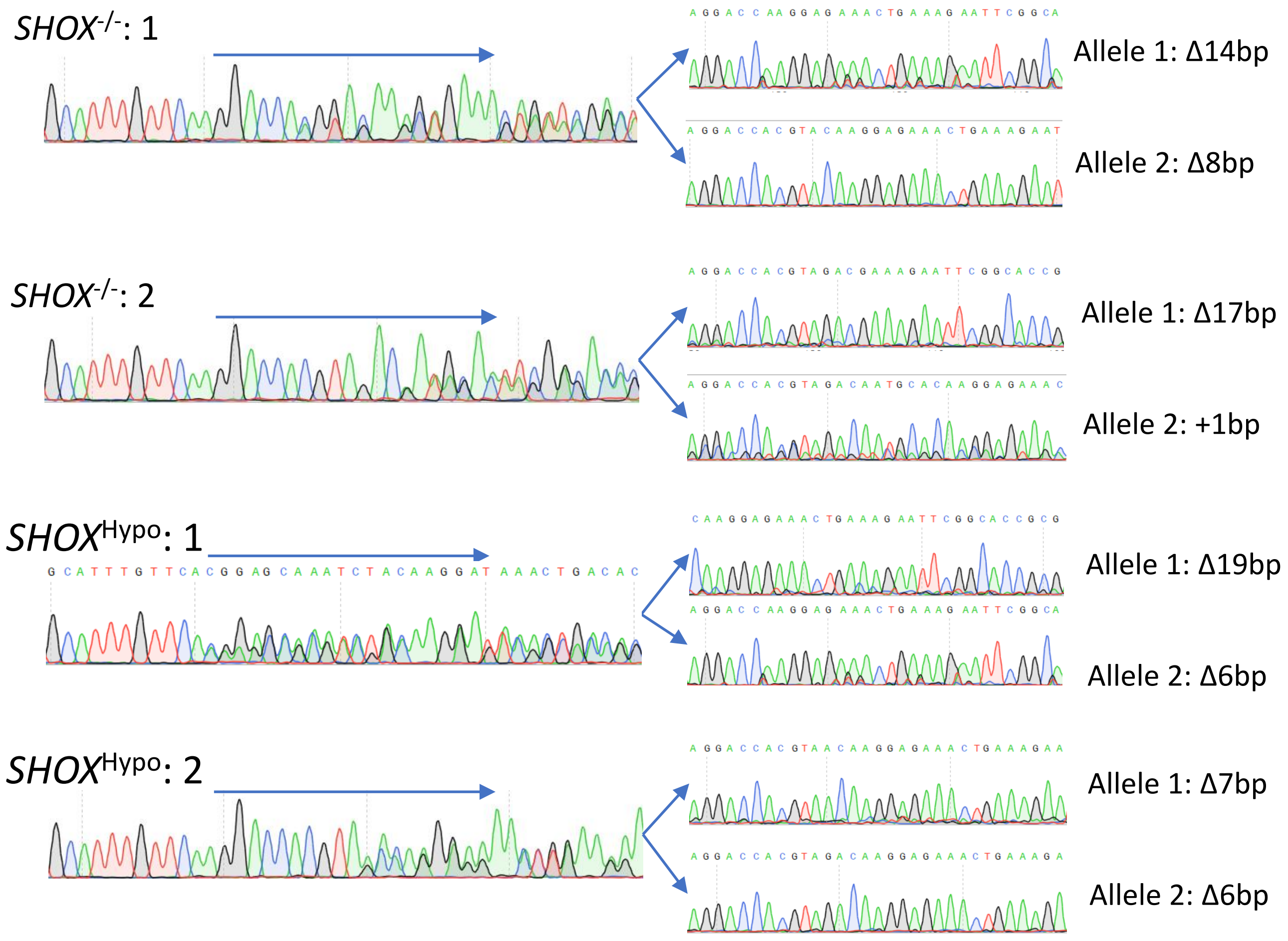

Additional file 1: Figure S3. Sequencing results of H1 *SHOX* clones

Sequencing chromatogram results of H1 *SHOX* mutant clones and plasmid containing SHOX PCR fragments of the clones showing mutations at gRNA site

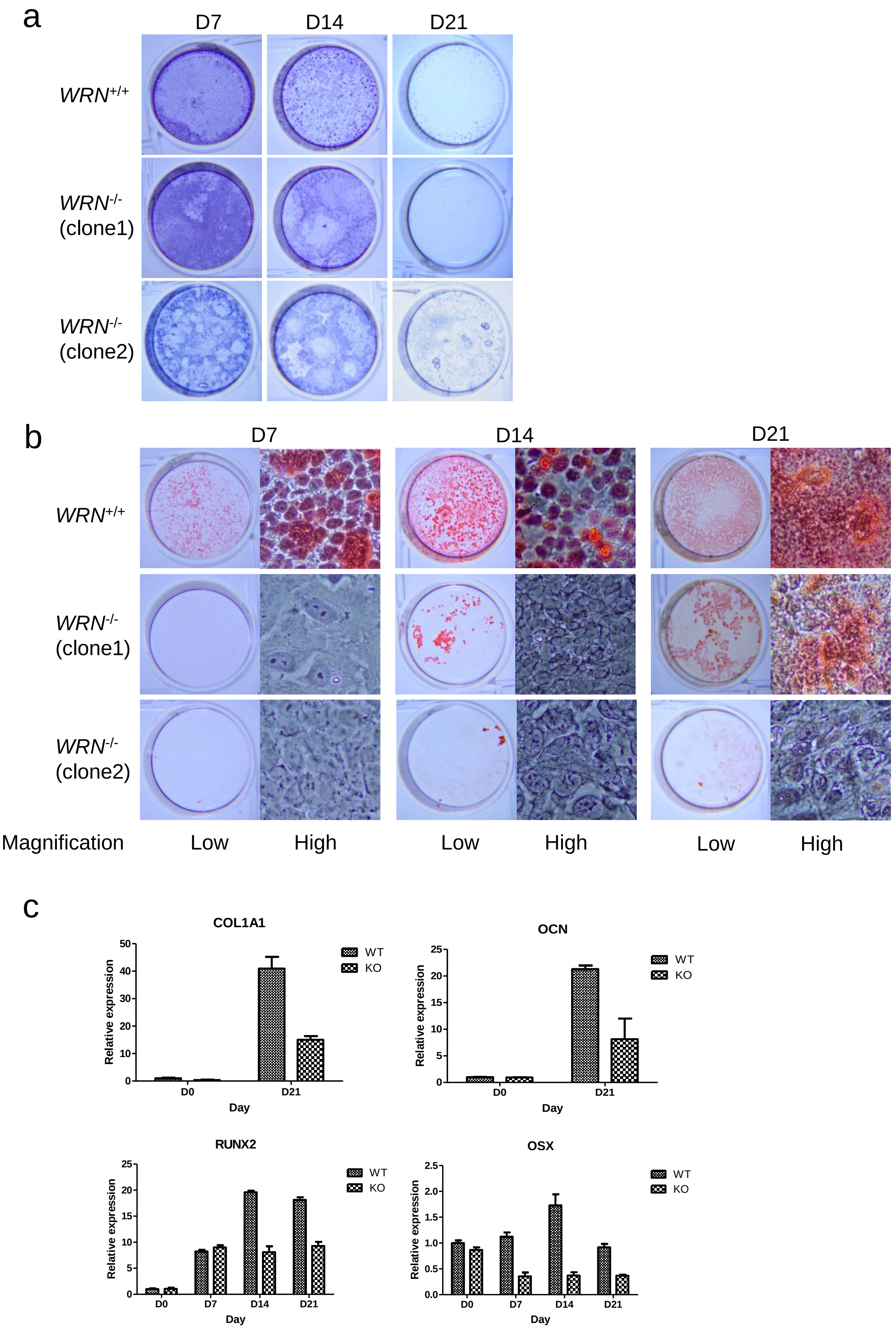

Additional file 1: Figure S4. Osteogenesis of *WRN*$^{+/+}$ and *WRN*$^{-/-}$ cells.
WRN$^{+/+}$ (WT) and *WRN*$^{-/-}$ (KO) hESCs were induced to differentiation to osteoblasts. Cells were characterized by (**a**) alkaline phosphatase staining, (**b**) Alizarin Red S staining at days 7, 14 and 21, respectively. (**c**) The expressions of osteogenic markers COL1A1, OCN, RUNX2 and OSX by RT-qPCR.
